# Genotyping of DPYD and UGT1A1 in Brazilian oncological patients: applying pharmacogenomics in an admixed population

**DOI:** 10.1590/1806-9282.20251174

**Published:** 2026-04-20

**Authors:** Vinicius Humberto Bandeira Cereser, Camila Motta Venchiarutti Moniz, Guilherme Suarez-Kurtz, Maria Del Pilar Estevez-Diz

**Affiliations:** 1 Faculdade de Medicina da Universidade de São Paulo – São Paulo (SP), Brazil.; 2 Faculdade de Medicina da Universidade de São Paulo, Instituto do Câncer do Estado de São Paulo – São Paulo (SP), Brazil.; 3 Instituto Nacional de Câncer – Rio de Janeiro (RJ), Brazil.

**Keywords:** Dihydropyrimidine dehydrogenase deficiency, UDP-Glucuronosyltransferase 1A1, Pharmacogenetics, Gastrointestinal neoplasms

## Abstract

**OBJECTIVE:**

The aim of this study was to determine the frequency of *UGT1A1* and *DPYD* polymorphisms among Brazilian patients with gastrointestinal cancer.

**METHODS:**

UGT1A1 and *DPYD* polymorphisms were selected due to their importance in the metabolism of fluoropyrimidines and irinotecan, and the existence of available clinical guidelines for dosing recommendations based on genetic testing. Polymorphism rs3918290, rs67376798, rs56038477, rs55886062, rs115232898, and rs887829 were investigated in 100 patients. Allelic discrimination was performed using the TaqMan Assay. Minor allele frequencies were calculated, and metabolic phenotypes were inferred based on the Clinical Pharmacogenetics Implementation Consortium and the Dutch Pharmacogenetics Working Group guidelines.

**RESULTS:**

For *DPYD*, rs56038477 (n=100) showed a minor allele frequency of 1.50% (CI 0.51–4.32), rs115232898 (n=49) had a minor allele frequency of 1.02% (CI 0.18–5.56), and rs3918290 (n=100) had a minor allele frequency of 0.50% (CI 0.09–2.78). No variants were observed in rs67376798 (n=100) or rs55886062 (n=44). A total of 4% of patients received a Clinical Pharmacogenetics Implementation Consortium *DPYD* activity score (AS) of less than 2.0, with 3% of 1.5 AS and 1% of 0.5 AS. For *UGT1A1*, rs887829 (n=100) revealed a minor allele frequency of 37.5% (CI 31.09–44.39) and 16% of patients with Dutch Pharmacogenetics Working Group poor metabolizer phenotype.

**CONCLUSION:**

The presence of *DPYD* and *UGT1A1* variants is considerable among Brazilian gastrointestinal cancer patients, reinforcing the promising use of pre-therapeutic genetic tests for irinotecan and fluoropyrimidines to avoid severe toxicities related to treatment. Genetic panels for these variants must be continuously updated and adapted for population-specific characteristics.

## INTRODUCTION

Pharmacogenetics benefits patient care by using a personalized approach to maximize therapeutic advantages while reducing adverse drug reactions^1^. Differences in phenotypes that affect tolerability to medications can be predicted through mutations in certain pharmacogenes, guiding the selection of better individualized treatments for patients with safety and cost-effectiveness^2^. The field has experienced significant implementation growth in the past decades, especially due to the cost decrease of multigene panels^3^ and the creation of international clinical guidelines such as the Clinical Pharmacogenetics Implementation Consortium (CPIC)^4^. This increase can be observed in the composition of the CPIC membership itself, which rose from just 60 members in 2012 to 781 members from 588 institutions in 2025^4^. Notably, American initiatives such as the "All of Us" Research Program from the National Institute of Health (NIH) and large biobanking programs also increased awareness and access to pharmacogenetics research^3^. Besides, in Europe, a remarkable increase in genetic testing requests and national guidelines was observed in multiple countries since pre-therapeutic *DPYD* genotyping was endorsed by the European Medicines Agency (EMA)^5,6^.

Meanwhile, most pharmacogenetics data suffer from the lack of ethnic diversity and come from studies of white European and North American populations^3,7-10^. This has significant implications as we consider their implementation in clinical practices worldwide, creating a barrier for widespread implementation due to its effect weakening the evidence for clinical validity necessary for progress of the field^3^. The Brazilian ­population is heavily heterogeneous, from the admixture among Europeans, Africans, and Indigenous groups, directly impacting the amount of deleterious genotypes observed in individuals^11^. This limits the extrapolation of international data and guidelines, upholding the need for studies that further examine the prevalence of polymorphisms of clinical significance in this population^12,13^.

Fluoropyrimidines and irinotecan are part of the main chemotherapy drugs for gastrointestinal cancers^5,14^. However, their use can still result in significant toxicities, especially among those patients with variants in the *DPYD* and *UGT1A1* genes due to their importance in the metabolism of these drugs^14,15^, justifying the existence of clinical guidelines with dosing recommendations based on genetic testing^2,5,12,14-16^.

Therefore, this study aims to assess the frequency of one polymorphism in *UGT1A1* and five different polymorphisms in *DPYD* among Brazilian oncological patients with gastrointestinal tumors. We hypothesized that the data from this interest group of oncological patients would align with the data observed in larger Brazilian non-oncological cohorts, demonstrating that it could be used to help guide the decision of the best polymorphisms to be used in the implementation of clinical pre-therapeutic tests for irinotecan and fluoropyrimidines in Brazil.

## METHODS

### Patients and sample

This study included 100 consecutive patients from a large public oncological hospital in São Paulo, Brazil. All patients had to be ≥18 years old with a diagnosis of a gastrointestinal tumor and be candidates for the use of fluoropyrimidines and/or irinotecan. All patients were genotyped for both *DPYD* and *UGT1A1*, and the use of both of the drugs at the time of enrollment was not required since the observance of clinical toxicity outcomes was not included in this study. No limitation for inclusion was made regarding the subtype of gastrointestinal tumor, time of diagnosis, cancer staging, or previous line of treatment. Patients had to be able to understand, agree to participate, and sign the informed consent form that was explained to them. Patients were excluded if they refused to sign the informed consent form or if a peripheral blood sample could not be obtained. After sample collection, an epidemiological questionnaire was applied.

### Polymorphism selection

The single-nucleotide polymorphisms (SNPs) chosen were based on the guidelines of the Clinical Pharmacogenetics Implementation Consortium (CPIC) and the Dutch Pharmacogenetics Working Group (DPWG).

For the *DPYD*, the initial study design comprehended a panel of four polymorphisms: rs3918290 (c.1905+1G>A DPYD*2A), rs67376798 (c.2846A>T *p.D949V*), rs56038477 (c.1236G>A *HapB3*), and rs55886062 (c.1679T>G DPYD*13). However, rs55886062 was not detected among initial participants or multiple Brazilian cohorts^17^. Thus, the panel was modified to substitute rs55886062 for rs115232898 (c.577A>G *p.Y186C*), which is linked with African ancestry^18^ and is more aligned with the composition of the Brazilian population. It is correlated with a 46% lower dihydropyrimidine dehydrogenase (DPD) activity in carriers^18^ and is reported to have a Minor allele frequency (MAF) of 1.30% among African Brazilians^9^.

For *UGT1A1*, rs887829 (NC_000002.12:g.233759924C>T, UGT1A1*80) was analyzed. It has a very high linkage disequilibrium with *28 and *37, and it can reliably be used to infer *UGT1A1* metabolizer status^19,20^. Besides, using UGT1A1*80 as an alternative to UGT1A1*28 could be a less expensive and easier to implement^20^, and the distribution of variants in UGT1A1*80 also does not seem to change across most Brazilian ethnic groups^12^.

### Genotyping

Dihydropyrimidine dehydrogenase (DNA) was extracted from the peripheral blood samples using QIAamp DNA Blood Midi (Qiagen, Germany) with peripheral leucocytes. Quantification and purity of the DNA were determined using a NanoDrop 1000 Spectrophotometer (Thermo Fisher Scientific, USA). The integrity of DNA was analyzed through 1% agarose gel electrophoresis. Tris-ethylenediaminetetraacetic acid buffer was used for DNA elution. After DNA extraction, allelic discrimination was performed using the TaqMan Assay (Applied Biosystems, USA).

### Phenotype inference

The metabolizer phenotypes were inferred based on the CPIC and DPWG guidelines. In the case of *DPYD*, analysis and dosing recommendations followed the CPIC's allele functionality table and genotype-phenotype tables, in which patients are attributed an Activity Score (AS) that ranges from 2.0 (normal metabolizers), 1.5 or 1.0 (intermediate metabolizers), and 0.5 or 0 (poor metabolizers)^15^. Regarding *UGT1A1*, the inferred metabolizer phenotype followed both the updated *UGT1A1* Diplotype-Phenotype Table from CPIC and the DPWG guidelines regarding *UGT1A1* phenotyping, considering the very high linkage disequilibrium between *80 and *28/*37 and that only *80 was genotyped in the samples^14,21^. Thus, heterozygous UGT1A1*80 was considered an intermediate metabolizer (IM), and homozygous UGT1A1*80 was considered a poor metabolizer. Therapeutic irinotecan dosing recommendations were established based on the DPWG guideline^14^.

### Statistical analysis

MAF were calculated by direct gene counting, with 95%CIs estimated through the Wilson score method. Hardy-Weinberg equilibrium (HWE) was assessed using the chi-square (χ^2^) test without continuity correction. For variants with very low frequencies (MAF<5%) or no observed variation (MAF=0), HWE was not tested due to limited statistical power or was considered not applicable. Statistical significance was set at p<0.05. Analyses were performed using the R software (version 4.5.0).

### Ethical approval

This study was approved by the Hospital das Clínicas da Faculdade de Medicina da Universidade de São Paulo Ethics Committee for Research Protocol Analysis, CAAE registration 23358719.3.2004.0068, and by the Instituto do Câncer do Estado de São Paulo (ICESP) Scientific Committee, CCEP ICESP registration 1984/21.

## RESULTS

DNA samples from 100 patients were analyzed (n=100). A questionnaire was applied after blood collection to compile the demographic and epidemiological data. One patient did not answer the questionnaire and therefore was not added to the results in Table 1 (n=99). The demographic analysis indicates the majority of patients declared themselves as part of an ethnicity other than white, with 43.4% declaring themselves as black or "pardo" (in Portuguese). All ethnic data were self-reported, following the approach adopted by the Brazilian Census. No genetic test was performed to attempt to determine patients’ genetic ethnic ancestry. No analysis was performed to distinguish the prevalence of the polymorphisms within the self-reported ethnic groups in this study due to the sample size of the ethnic groups not allowing for simultaneous observation of all the polymorphisms of interest. The sample was balanced between male and female participants (50.5–49.5%). Data regarding some comorbidities that are part of the study's questionnaire were included in Table 1 since the use of pharmacogenomics has the potential of being especially beneficial for patients with coexisting chronic clinical conditions or who take multiple medications^10^.

**Table 1:** Demographic and epidemiological characteristics of the patient population (n=99).

Mean age—years		59.39
Median age (min–max)—years		59 (27–87)
Male sex—n (%)		50 (50.5)
Female sex—n (%)		49 (49.5)
Ethnicity—n (%)		
	White	47 (48.5)
	"Pardo"*	29 (29.3)
	Black	14 (14.1)
	Asian	1 (1.0)
	Other	8 (8.1)
Family history of any cancer—n (%)		
	Yes	76 (76.8)
	No	23 (23.2)
Presence of other comorbidities—n (%)		
	Diabetes mellitus	7 (7.1)
	Arterial hypertension	29 (29.6)
	Chronic obstructive pulmonary disease	5 (5.1)
	Hypercholesterolemia	9 (9.3)

Min: minimum; max: maximum.

* "Pardo" (in Portuguese) refers to the official Brazilian Census category, which includes individuals of mixed European, African, and Indigenous ancestry.

Table 2 summarizes the genotype frequencies and MAFs of the SNPs. For *DPYD*, the most observed polymorphism was in rs56038477 (n=100), with a MAF of 1.50 (CI 0.51–4.32), followed by rs115232898 (n=49) with a MAF of 1.02 (CI 0.18–5.56) and rs3918290 (n=100) with a MAF of 0.50 (CI 0.09–2.78). No variants were observed in rs67376798 (n=100) or rs55886062 (n=44). Regarding *UGT1A1*, rs887829 (n=100) analysis revealed 16% of patients possessed a variant homozygous genotype (*80/*80) and a MAF of 37.5% (CI 31.09–44.39), with distribution consistent with HWE (p=0.4084).

**Table 2:** Genotype frequencies and minor allele frequencies of selected single-nucleotide polymorphisms in *DPYD* and *UGT1A1*.

Gene	SNP	Tested—n	WT homozygous—n (%)	Heterozygous—n (%)	Variant homozygous—n (%)	MAF—% (95%CI)	HWE p-value
*DPYD*	rs56038477 (c.1236G>A *HapB3*)	100	97 (97.0%)	3 (3.0%)	0 (0.0%)	1.50 (0.51–4.32)	Not tested (low MAF)
	rs3918290 (c.1905+1G>A DPYD*2A)	100	99 (99.0%)	1 (1.0%)	0 (0.0%)	0.50 (0.09– 2.78)	Not tested (low MAF)
	rs67376798 (c.2846A>T *p.D949V*)	100	100 (100.0%)	0 (0.0%)	0 (0.0%)	0.00 (0.00–1.88)	Not applicable
	rs115232898 (c.577A>G *p.Y186C*)	49	48 (97.96%)	1 (2.04%)	0 (0.0%)	1.02 (0.18–5.56)	Not tested (low MAF)
	rs55886062 (c.1679T>G DPYD*13)	44	44 (100.0%)	0 (0.0%)	0 (0.0%)	0.00 (0.00–4.18)	Not applicable
*UGT1A1*	rs887829 (UGT1A1*80)	100	41 (41.0%)	43 (43.0%)	16 (16.0%)	37.50 (31.0–44.39)	0.4084

SNP: single-nucleotide polymorphism; WT: wild-type; MAF: minor allele frequency; CI: confidence interval; HWE: Hardy–Weinberg equilibrium.

Phenotype distributions are expressed in Table 3, accompanied by the guideline therapeutic dosing recommendations. For *DPYD*, 4 (4%) patients were assigned an activity score of less than 2.0. One participant received an AS of 0.5 due to having variants on both rs3918290 (variant of no function) and rs56038477 (variant of decreased function), receiving a recommendation to avoid use of 5-fluorouracil or 5-fluorouracil prodrug-based regimens. The other three patients were assigned an AS of 1.0 due to having either a variant of rs56038477 (variant of decreased function) or rs115232898 (variant of decreased function). As for *UGT1A1*, 41 (41%) were regarded as normal metabolizers (NM), while 43 (43%) were considered IMs, and 16 (16%) patients were inferred as poor metabolizers (PMs), with a recommended 30% reduction in the usual irinotecan initial dose.

**Table 3:** Phenotype distribution of *DPYD* and *UGT1A1* in study participants (n=100).

Gene	Phenotype category	n (%)	Guideline therapeutic dosing recommendation
*DPYD*	AS=2.0	96 (96%)	CPIC: Use label recommended dosage and administration.*
	AS=1.5	3 (3%)	CPIC: Reduce dose by 25–50%.*
	AS=1.0	0 (0.0%)	CPIC: Reduce dose by 50%.*
	AS=0.5	1 (1%)	CPIC: Avoid use of 5-fluorouracil or 5-fluorouracil prodrug-based regimens.*
	AS=0.0	0 (0.0%)	CPIC: Avoid use of 5-fluorouracil or 5-fluorouracil prodrug-based regimens.*
*UGT1A1*	Normal metabolizer	41 (41%)	DPWG: No action required.**
	Intermediate metabolizer	43 (43%)	DPWG: No action required.**
	Poor metabolizer	16 (16%)	DPWG: Start with 70% of the normal irinotecan dose.**

AS: activity score; CPIC: Clinical Pharmacogenetics Implementation Consortium; DPWG: Dutch Pharmacogenetics Working Group.

* See Amstutz et al.^15^ for details.

** See Hulshof et al^14^ for details.

## DISCUSSION

The findings reinforce the relevance of implementing pharmacogenetics testing in clinical practice and the importance of furthering research on the distribution of variants in admixed populations. Data from European and North American groups might steer genotyping panels to include SNPs more frequently seen in homogeneous white groups, differing from populations with strong black and indigenous ancestry.

The MAFs of *DPYD* variants observed align with previous findings in Brazilian populations. Two large studies in Brazil were used for comparison: one with 1.171 elderly individuals^22^ and another with 800 healthy Southern Brazilians^8^. For rs3918290, our MAF was 0.50% (CI 0.09–2.78), higher than the 0.25%^8^ and 0.13%^22^ from the other groups. While for rs67376798, our MAF was 0.00 % (CI 0.00–1.88), smaller than the equal MAFs of 0.38% observed in both studies^8,22^. The biggest difference was observed in rs56038477: our MAF was 1.50% (CI 0.51–4.32), compared to 0.11%^8^ and 0.43%^22^. Regarding rs115232898, our MAF of 1.02% (CI 0.18–5.56) conformed with the 2.6% observed in the study on elderly individuals^22^ and the 1.3% reported in another study specifically conducted on 115 healthy Brazilian individuals of African ancestry^9^, while the group of 800 healthy subjects did not analyze this SNP^8^. It is important to note that no variants in rs55886062 were observed either in our study or in the larger ones, reinforcing that this variant might not be essential in tests for Brazilian populations. In a context of limited resources for genetic testing, it is relevant to consider the change in rs55886062 for more prevalent variants, such as rs115232898. The link of rs115232898 with African ancestry and its impact on DPD activity in carriers^18^ makes it a great candidate for inclusion in Brazilian *DPYD* pre-therapeutic genetic panels^17^.

As demonstrated, even in this 100-participant group of gastrointestinal cancer patients, three received recommendations to start with only 50% of standard dosing for fluoropyrimidines, and one received the recommendation to completely avoid these medications due to the risk of severe adverse drug reactions. Despite their relatively low frequencies, the clinical impact of these variants should be considered when prescribing fluoropyrimidine-based therapies, since toxicities associated with them can be life-threatening and burden patients and the healthcare systems^16^. A growing number of medical societies recommend routine pre-therapeutic genetic testing of *DPYD*, as the European Medicines Agency, with reports of an increasing number of genotype and phenotype testing and adoption of novel local clinical guidelines across Europe^5,6^.

Remarkably, rs887829 (*UGT1A1*) presented with a MAF of 37.5% (CI 31.09–44.39) in our research, consistent with findings from multiple other studies of the Brazilian population^12,19,22^. The same project with 1.171 elderly Brazilian individuals found for rs887829 a MAF of 35%^22^, and analysis of the distribution of this variant among different Brazilian ethnic groups indicated MAFs of 35.4% (black), 39.4% ("pardo"), and 36.9% (white)^12^.

A recent analysis by Perini et al. of the distribution of *UGT1A1* polymorphisms among indigenous groups in the Brazilian Amazon demonstrated a MAF of 44.1% among Yanomami (n=92) and 47.0% for Paiter-Suruí (n=88) regarding rs887829^19^. The same study compared the CPIC *UGT1A1* inferred metabolizer status for these groups with other studies in non-indigenous Brazilians. It observed a prevalence of 20.5% of PM and 55.4% of IM among Yanomami, and 18.8% of PM and 50.6% of IM for Paiter-Suruí, while studies of non-indigenous Brazilians presented a variation of 10–13% for PM and 41–43% for IM^19^. Our study observed 16% of PM and 43% of IM, indicating our findings align with the literature. This high frequency of PM reinforces the need for implementing pre-therapeutic *UGT1A1* testing, with existing cost-effectiveness reports for routine testing due to reductions in severe neutropenia and hospitalizations^16^.

Unfortunately, the adoption of pharmacogenetics is still incipient as an oncological routine in developing countries like Brazil, especially within the Public Healthcare System ("Sistema Único de Saúde," in Portuguese)^23^. Pioneer implementation projects within the public sector have only been able to successfully execute these tests for patients as part of research studies due to many factors, but especially the need for more evidence of cost-effectiveness^23^. Despite the existence of pre-therapeutic genetic testing for chemotherapy drugs in the private sector in Brazil, its adoption is not widespread even in this context^23^. Even in the United States, there are still several implementation challenges for the use of these tests, such as a lack of awareness of positive patient experiences, clinician education, clinician perception of benefits of pharmacogenomics, inadequate allele coverage in panels, lack of Electronic Medical Record (EMR) integration, and reimbursement issues with insurance companies^10^.

### Limitations

The study's main limitation is its relatively modest sample size, restricting its ability to detect very rare variants. The differences in MAFs observed could be a direct result of it, considering the values observed in other studies are almost entirely within our 95%CI. Other limitations include that phenotypes are exclusively inferred rather than experimentally determined for comparison, and the absence of clinical outcomes to contrast metabolizer status with the occurrence of toxicities. Besides, this study did not follow how many patients effectively had their treatment modified to adhere to the recommendations. Patients had their guideline recommendations added to their EMR so that their physicians could make individualized decisions regarding their application in the clinical setting.

## CONCLUSION

The data from this oncological patient population aligns with the observed data from other larger studies in the Brazilian population, in accordance with our initial hypothesis. The presence of these *DPYD* and *UGT1A1* variants is considerable among Brazilian gastrointestinal cancer patients, and the use of pre-therapeutic genetic testing in this context is growing as a promising precision medicine tool to avoid severe toxicities. Additionally, genetic panels for these variants must be continuously updated and adapted for population-specific characteristics, incorporating new findings about clinically significant variants and genetic diversity in admixed populations.

## Data Availability

The datasets generated and/or analyzed during the current study are available from the corresponding author upon reasonable request.
